# The International Medical Annual and Practitioner’s Index

**Published:** 1891-07

**Authors:** 


THE INTERNATIONAL MEDICAL ANNUAL AND PRACTITIONER'S INDEX. A Work of Reference for Medical Practitioners 1891. Edited by PERCY WILDE, M. D. Series of Medical Classics. E. B. Treat, publisher, 5 Cooper Union, New York. Pp. 580. Price, $2.75.

The index to this volume contains nearly three thousand references to diseases and remedies. The literature of the year has been carefully analyzed and the practical and useful has been happily condensed, and is here presented in admirable form. Although special attention has been paid in setting forth the therapeutic side of medicine, pathology and diagnosis have not been slighted. We can scarcely conceive how any physician can do without an Annual of the progress of medicine, and we know of none better than Treat's. The corps of editors comprises representative men of the profession in America, England, France, and India. Besides the references, this volume contains some interesting and valuable articles, among which may be mentioned: "The Hand as a Diagnostic Feature in Diseases of the Nervous System," by Dr. E. L. Fox; "Methods of Testing for Errors of Refraction," by Mr. William Lang; "Recent Tests for Deafness," by Dr. J. Dundas

Grant; "The Sputa as a Means of Diagnosis," by Dr. F. J. Wethered; "The Motor-Centers of the Brain," by Mr. W. H. Elam; "The Diagnosis of Functional and Organic Diseases of the Heart," by Drs. Leaming and Jackson, of New York, and "The Diagnosis of Gastric Neurasthenia," by Dr. Dujardin–Beaumetz, of Paris.

The appearance of this book is similar to the others of this series, containing many instructive engravings, which add much to its usefulness. W. C. K.

